# Using claws to compare reproduction, stress and diet of female bearded and ringed seals in the Bering and Chukchi seas, Alaska, between 1953–1968 and 1998–2014

**DOI:** 10.1093/conphys/coaa115

**Published:** 2021-01-06

**Authors:** Danielle D Crain, Shawna A Karpovich, Lori Quakenbush, Lori Polasek

**Affiliations:** 1 Department of Biology, Baylor University, Waco TX, 76706, USA; 2 Alaska Department of Fish and Game, 1300 College Road, Fairbanks, AK 99701, USA; 3 Alaska Department of Fish and Game, 1255 W 8^th^ St, Juneau, AK 99802, USA

**Keywords:** bearded seals, claws, cortisol, pregnancy, ringed seals, stable isotopes

## Abstract

Rapid climate warming is decreasing sea ice thickness, extent and duration. Marine mammals such as bearded (*Erignathus barbatus*) and ringed (*Pusa hispida*) seals, which use sea ice for pupping, molting and resting, may be negatively affected. Claws from bearded and ringed seals store up to 14 and 12 years of sequential analyte data, respectively. These data can be used to compare reproduction, stress and diet across decades. In this study, we compare progesterone, cortisol and carbon and nitrogen stable isotopes in female bearded and ringed seals during 1953–1968 (pre-1968, a period prior to sea ice decline) to 1998–2014 (post-1998, a period during sea ice decline). When comparing these periods, bearded seals had statistically higher cortisol concentrations post-1998, and for both species δ^13^C was more negative post-1998, while progesterone and δ^15^N did not change. There was a positive relationship between progesterone and cortisol Z-scores for both species, except for ringed seals post-1998. There was a negative relationship between cortisol Z-scores and δ^13^C for bearded seals evident in post-1998 indicating that higher cortisol Z-scores are associated with more negative δ^13^C in bearded seals in recent years. This negative relationship between cortisol and δ^13^C in bearded seals suggests a shift to higher prey diversity, possibly due to changes in sea ice in the Pacific Arctic evident post 1998. Progesterone Z-scores corresponded to expected differences among non-pregnant, unimplanted, implanted and post-partum individuals. Using these data, pregnancy history was determined for reproductive years for each individual female sampled, which could allow for yearly pregnancy rates to be calculated given a large enough representative sample of the population. These results combine decades of observational studies with hormones and stable isotopes to infer changes in reproduction, stress and diet, as well as the connection between these life history parameters.

## Teaser

We investigate the relationship among reproduction, stress and diet in bearded and ringed seals by measuring hormones and stable isotopes stored in claws. As a result, we calculated pregnancy rate of individual females and found that ringed seals seem able to adjust well to changes in diet, unlike bearded seals.

## Introduction

Climate warming is disproportionately affecting the Arctic, causing decreased sea ice thickness, coverage and duration (Stroeve *et al.* 2007; Frey *et al.* 2015; Kwok 2018; Wang *et al.* 2018; Shaftel *et al.* 2020), which has the potential to affect species that rely on sea ice for some life history events (e.g. pupping and molting; Moore and Huntington 2008; Cameron *et al.* 2010; Kovacs *et al.* 2011). Two species that rely on Arctic sea ice are bearded (*Erignathus barbatus*) and ringed (*Pusa* [*Phoca*] *hispida*) seals, which have been studied for more than 60 years in Arctic Alaska (Burns 1981; Frost and Lowry 1981; Quakenbush *et al.* 2011a, 2011b; Crawford *et al.* 2015). Both species were listed as threatened under the Endangered Species Act in 2012 because declines in sea ice over the next century were predicted to cause populations to decline (U.S. Federal Register 2012a, 2012b). Bearded seals associate with broken, drifting pack ice, and pups are born on top of the ice (Burns 1981). Their population size is not well known, but a conservative estimate for the US waters of the Bering and Chukchi seas is >357 328 (Nelson *et al.* 2019); there is no information on trend. Ringed seals are associated with shorefast ice where they maintain breathing holes and create subnivean lairs in which they rest and pup (Smith and Stirling 1975; Frost and Lowry 1981). Ringed seals are more abundant than bearded seals and a conservative estimate for the US portion of their range is >470 000 with no information on trend (Nelson *et al.* 2019). Both species, once mature, breed annually and maintain relatively high pregnancy rates with bearded seals exhibiting >90% pregnancy rate and ringed seals with more variable >75% pregnancy rates (Frost and Lowry 1981; Crawford *et al.* 2015). However, these seals differ in their diets: bearded seals are benthic feeding generalists and ringed seals are pelagic feeding generalists (Burns 1981; Frost and Lowry 1981). Therefore, bearded and ringed seals may respond differently to changes in Arctic sea ice.

Bearded and ringed seal diet (from stomach contents), body condition and reproductive rate has been compared between historical data (1975–1984; before sea ice decline) and recent data (2003–2012; after sea ice decline; Crawford *et al.* 2015). No comparison has yet been made between historical and recent ice seal data using hormone biomarkers (e.g. progesterone and cortisol). Hormone biomarkers of reproduction and stress are extremely useful in studies of the life history of long-lived and migratory animals. Progesterone is secreted by the corpus luteum (CL) after ovulation and, if pregnancy occurs, the CL will continue to secrete progesterone until the developing placenta takes over. Progesterone has been used to determine pregnancy in many mammalian species, including seals (Henricks *et al.* 1970; Stabenfeldt *et al.* 1970; Raeside and Ronald 1981; Sawyer-Steffan *et al.* 1983; Reijnders 1990; Boyd 1991; Gardiner *et al.* 1996; Greig *et al.* 2007; Trego *et al.* 2013; Zhang *et al.* 2014). Some mammalian species, like bearded and ringed seals, exhibit embryonic diapause, where the blastocyst is not implanted into the uterine lining for several months (Renfree and Fenelon 2017). Therefore, the presence of a CL indicates ovulation, but cannot distinguish diapause, active implantation or pseudopregnancy (Renfree and Fenelon 2017). Animals in embryonic diapause (i.e. unimplanted) and ovulating animals can exhibit highly variable progesterone concentrations (Renfree and Fenelon 2017). This makes it difficult to distinguish implanted individuals from unimplanted or ovulating individuals until late gestation when the progesterone levels are generally higher, facilitating the comparison of pregnant to non-pregnant seals (Gardiner *et al.* 1996). Cortisol is a glucocorticoid frequently measured in the serum, urine, feces and blubber of pinnipeds (Gardiner and Hall 1997; Hunt *et al.* 2004; Constable *et al.* 2006; Kershaw and Hall 2016; Hall *et al.* 2020). It is excreted by the adrenal glands in response to physical and/or psychological stress through activation of the hypothalamic–pituitary–adrenal (HPA) axis, which is why it is often termed a ‘stress’ hormone (Sapolsky *et al.* 2000; MacDougall-Shackleton *et al.* 2019). Cortisol plays many roles as a result of HPA activation, including gluconeogenesis, increased mobilization of energy to muscle, decreased digestion and decreased energy toward reproduction (Sapolsky *et al.* 2000; MacDougall-Shackleton *et al.* 2019).

Very few studies have combined hormone biomarker measurements with carbon and nitrogen stable isotope measurements (for review see Fleming *et al.* 2018). Stable isotope composition changes in the bodies of animals due to migration, varying diet, habitat and physiology (Newsome *et al.* 2010). Specifically, carbon and nitrogen stable isotopes can reveal changes in diet, where carbon can reveal changes in benthic/in-shore prey, pelagic/off-shore prey or increases in prey diversity; nitrogen can reveal changes in trophic level and denitrification/nitrogen fixation (Newsome *et al.* 2010; Fleming *et al.* 2018). Therefore, analyzing progesterone, cortisol and carbon and nitrogen stable isotopes in bearded and ringed seals would allow researchers to combine measurements related to reproduction, stress and diet of these seals.

**Table 1:** Claw samples from female bearded and ringed seals by region, specific region and period

Species	Period	Region	Total per region	Specific region
Bearded seal	Pre-1968	Bering	12	Gambell (9), Savoonga (3)
		Chukchi	0	NA
	Post-1998	Bering	8	Gambell (8)
		Chukchi	5	Point Hope (4), Shishmaref (1)
Ringed seal	Pre-1968	Bering	0	NA
		Chukchi	11	Nome (8), Little Diomede (2), Wainwright (1)
	Post-1998	Bering	3	Gambell (2), Hooper Bay (1)
		Chukchi	8	Shishmaref (7), Utqiagvik (1)

Front flipper claws from bearded and ringed seals archive hormones and stable isotopes deposited at the time of growth (Carroll *et al.* 2013; Karpovich *et al.* 2020); similar to pinniped whiskers (Karpovich *et al.* 2018), baleen (Hunt *et al.* 2014) and baleen whale earplugs (Trumble *et al.* 2013, 2018; Crain *et al.* 2020). Furthermore, bearded and ringed seal claws form light and dark bands that together correspond to one year of claw growth, traditionally being used to ascertain minimum age of the individual (McLaren 1958; Benjaminsen 1973; Ferreira *et al.* 2011). Hormones and stable isotopes, once deposited, remain stored long-term in the claw for decades without degradation (Carroll *et al.* 2013; Karpovich *et al.* 2020). To examine hormones and stable isotopes in samples from pre-1968 and post-1998, bearded and ringed seal claws were analyzed from female seals harvested in the Bering and Chukchi seas. Here, we present progesterone, cortisol, carbon and nitrogen stable isotopes from these periods to assess the changes in bearded and ringed seal reproduction, potential stress load and diet as well as comparing these parameters between bearded and ringed seals.

## Methods

**Figure 1:**
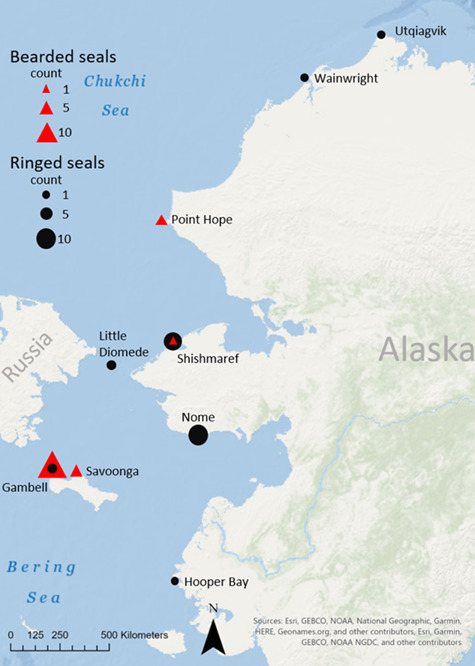
Harvest regions and specific regions for female bearded and ringed seals for which claws were analyzed

**Figure 2:**
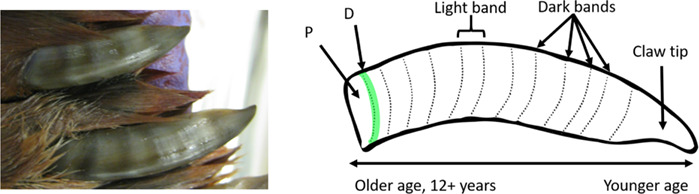
Bands in the claws of bearded and ringed seals are produced over time; light bands are produced in the late spring to late summer and the dark bands are produced in early fall to early spring (McLaren 1958; Benjaminsen 1973; Karpovich *et al.* 2020). The closer to the tip of the claw, the younger the age of the seal the claw band represents. In this example P indicates the light claw band that was forming prior to death in summer, and D (highlighted in green) represents the first dark band immediately distal to P. Photo used with permission from Sara Carroll, MS.

In this study, front flipper claws were used from 25 female bearded seals and 22 female ringed seals over two periods, 1953–1968 (pre-1968) and 1998–2014 (post-1998) from multiple locations in the Bering and Chukchi seas (Table 1, Fig. 1). Claws are one of many samples donated by Alaska Native subsistence hunters from their ice seal harvests to the Alaska Department of Fish and Game, which were later transferred to the University of Alaska Museum of the North. Specifically, these periods were chosen to represent before and after significant sea ice decline, which is predicted to affect ice seals (Cameron *et al.* 2010; Kelly *et al.* 2010). Bands on the claws of ice seals are believed to represent seasonal growth; in the Northern hemisphere: summer (light bands, April–July) and winter (dark bands, August–March; McLaren 1958; Benjaminsen 1973). In our study, it was not always clear by color alone if a band was deposited in the summer or winter. However, dark bands were always more narrow than neighboring bands and associated with a raised ridge, which was visually and texturally elevated. Therefore, using the ridges to identify dark bands allowed them to be consistently identified for sampling. Claw samples were processed using the same methods as in Karpovich *et al.* (2020). Briefly, claws were soaked in deionized water to reveal the claw bands and, using a Dremel® tool, all dark bands were sampled once and all light bands were sampled twice (left and right halves when examining Fig. 2) except in the case of the light band growing at the time of harvest (the most proximal band) which was only sampled once due to low sample mass of the currently growing band. Samples from ringed seals were further processed for 15 minutes using a Retsch® Mixer Mill MM400 due to uneven homogenization using the Dremel® tool alone. For both species, 5 mg of claw powder was extracted in 100% ACS grade methanol and centrifuged for 13 min at 10500 G force and 10°C. The supernatant was stored at ≤ −20°C until hormone analysis. Progesterone and cortisol hormone assays were validated via parallelism and accuracy tests for bearded and ringed seal claws previously, as well as hormone extraction efficiency comparing two homogenization methods (Karpovich *et al.* 2020).

Each seal was aged by enumerating the dark claw bands, providing a minimum estimate for age as annuli wear off on the distal end (at the claw tip; Fig. 2). Some individuals post-1998 were also aged by a lower canine tooth at Matson’s Laboratory (Milltown, Montana, USA), and the annual growth layers of the cementum were counted for a whole life age estimate (Fig. S1). Therefore, seals aged only by claw bands will have a younger mean age than seals aged by teeth if they are older than *ca*. 10 years.

Progesterone and cortisol for each seal’s entire claw (representing timespans of 4–14 years; Fig. 2) were Z-score normalized to facilitate comparison across age or calendar year for multiple individuals of the same species (Crain *et al.* 2020), because individual animals will often express different lifetime hormone means (Charapata *et al.* 2018; Trumble *et al.* 2018; Crain *et al.* 2020). A Z-score of negative one (−1) represents a data point one standard deviation below the mean, where the mean has a Z-score of 0 and any values above or below this are proportional to the standard deviation. Furthermore, Z-score normalization of hormone concentrations can also correct for hormone concentrations derived from different methodologies (Crain *et al.* 2020), thereby allowing for the comparison of Z-score normalized hormones in this study. Dark and light bands representative of one year were often combined to produce enough material for sufficient binding in the cortisol hormone assay. Therefore, for comparisons in a mixed methods framework, progesterone, δ^15^N and δ^13^C were also averaged over the same bands, after which Z-scores were re-calculated for progesterone. Using the *lme* function and *summary()* for the model from the nlme package in R (Pinheiro *et al.* 2018; R Core Team 2018), we compared the hormones and stable isotopes from pre-1968 to post-1998, controlling for tooth/claw age, individual seal and autocorrelation in a mixed model framework. Akaike information criterion (AIC) model selection was used for choosing statistically significant predictors. Grubbs’ test was used to identify outliers when appropriate.

Carbon and nitrogen stable isotope analyses were conducted on claw powder samples by the Alaska Stable Isotope Facility at the University of Alaska Fairbanks’s Water & Environmental Research Center. Stable isotope data was obtained using continuous-flow isotope ratio mass spectrometry. This method utilizes a Thermo Scientific Flash 2000 elemental analyzer and Thermo Scientific Conflo IV interfaced with a Thermo Scientific DeltaV^Plus^ Mass Spectrometer. Stable isotope ratios were reported in δ notation as parts per thousand (‰) deviation from the international standards VPDB (carbon) and air (nitrogen). Typically, instrument precision is < 0.2 ‰. Carbon stable isotopes were Suess corrected to the Bering Sea and the earliest year represented by these data (1953) to account for increased fractionation of carbon due to increased use of fossil fuels from 1850 to present day (Clark *et al.* 2019). Methods for Suess correction were the same as in Clark *et al.* (2019).

Once female seals are mature, breeding occurs annually and both species have relatively high pregnancy rates (Frost and Lowry 1981; Crawford *et al.* 2015). Reproductive status at time of death was available for 41 of the 47 seals in this study. Each ovary was inspected for a CL and corpora albicantia (CA), and the uterine horns were inspected for a fetus. If a seal was harvested before implantation occurred, pregnancy status could not be assessed. If a CL was present, but no fetus was visible, the seal was considered unimplanted. An unimplanted female could have a blastocyst which has not yet implanted, be pseudopregnant which includes ovulation and conservation of a CL without fertilization (Renouf *et al.* 1994), or could include females that did not implant at the end of diapause. Therefore, not all females with a CL necessarily become pregnant. Implanted individuals, or seals in active gestation, had a fetus present. Post-partum was indicated by the presence of a CL changing to a CA and a stretched uterine horn with a fresh placental scar. Non-pregnant females had no CL or fetus but should have a CA from the previous year. The reproductive tracts of two ringed seals, sampled in the fall of 2011, had stretched, thick uterine horns suggesting past reproductive activity, but the ovaries lacked both CL and CA (indicating no ovulation or reabsorption of CA as in Dabin *et al.* 2008); regardless, these females were not pregnant and for the purposes of this study assigned a reproductive status of anovulatory. Females were nulliparous (immature reproductive tracts with no signs of ovulation), primiparous (CL but no CA suggesting first ovulation) or multiparous (ovarian and uterine scarring). Longitudinal progesterone concentrations across the entire length of ringed seal claws were physiologically validated for pregnancy previously (Karpovich *et al.* 2020). Therefore, to examine how progesterone behaves in longitudinal claw samples of individual bearded and ringed seals of known reproductive status at the time of harvest, we focused primarily on the most recent bands: the most proximal band (P) and the band immediately distal to P (D; Fig. 2).

A one-way ANOVA was performed using the function *aov* followed by Tukey multiple pairwise-comparisons (function *TukeyHSD*) to assess how progesterone Z-score related to pregnancy stage and how pregnancy rate varied by period (R Core Team 2018). Pregnancy stage was determined by assessing both season the seal was harvested and its pregnancy status at its time of death. Bearded and ringed seals give birth and become pregnant in spring, have a three-month-long embryonic diapause in summer and implantation occurs in fall (Burns 1981; Frost and Lowry 1981). Seasons were assigned as follows: August–November as fall (seals are non-pregnant, in diapause or active gestation), December–March as winter (seals are non-pregnant or in active gestation), April–May as spring (non-pregnant, parturition, ovulation and/or breeding) and June–July as summer (non-pregnant or in diapause; Fig. S2). Unimplanted seals harvested in summer were considered to be in ‘early’ pregnancy. Implanted seals harvested in fall or winter were considered to be in ‘mid-’ pregnancy. Finally, hormone deposition in claws of implanted seals with fetuses present (spring) or post-partum (summer) seals were considered to be representative of ‘late’ pregnancy.

Pregnancy rate for individual females was measured as the number of pregnancies during the reproductive period of a female. The number of pregnancies during the reproductive period represented by the claw was calculated by summing one light band per year that had a progesterone Z-score above 0 (because 0 was the average progesterone Z-score for an unimplanted female seal). Using only one light band per year eliminated the chance of counting more than one pregnancy per year of claw growth. Minimum reproductive period was calculated by summing the number of years after the year in which the seal was first determined to be pregnant using progesterone Z-scores in the claw to the year of harvest. Due to the method of calculating pregnancy rate, primiparous individuals and individuals harvested at age four or younger were not included in this analysis. A one-way ANOVA using the function *aov* (R Core Team 2018) was used to compare pre-1968 and post-1998 individual pregnancy rates.

## Results

Bearded and ringed seal claws in this study represented a mean of 9.2 ± 2.3 years, with no difference by species or year (Student’s *t*-test, *P* > 0.05). No difference in progesterone or cortisol was detected between the Bering and Chukchi seas for either species post-1998. No such comparisons could be made for pre-1968 due to lack of samples in either the Chukchi or the Bering seas during that period (Table 1).

### Pre-1968 compared to post-1998

**Figure 3:**
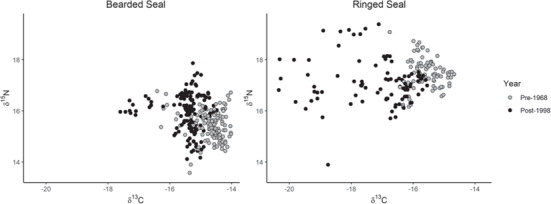
δ^13^C (Suess corrected) and δ^15^N stable isotope ratios by period (pre-1968 and post-1998) for female bearded and ringed seal claws by band

There was no difference in progesterone concentration between species from the pre-1968 to post-1998 (F = 0.45, *P* > 0.05 and F = 0.02, *P* > 0.05, respectively; Table 2). However, there was a significant increase in cortisol concentration over the same time frame for female bearded seals (F = 9.7, *P* < 0.01), but not for female ringed seals (F = 3.8, *P* > 0.05; Table 2). There was a significant difference in δ^13^C for pre-1968 compared to post-1998 with bearded and ringed seal δ^13^C becoming more negative (F = 11.2, *P* < 0.05 and F = 19.4, *P* < 0.05, respectively; Table 3). There was no difference in δ^15^N for pre-1968 compared to post-1998 for bearded or ringed seals (F = 3.9, *P* > 0.05 and F = 0.0, *P* > 0.05, respectively; Fig. 3, Fig. S3). One bearded seal and two ringed seals had a significantly more negative mean δ^13^C when compared to their post-1998 counterparts (Grubbs’ test: *P* < 0.001 and *P* = 0.05, respectively) and one ringed seal had a significantly higher mean δ^15^N than post-1998 ringed seals (Grubbs’ test: *P* = 0.003), identifying these individuals as outliers.

**Table 2:** Cortisol and progesterone (mean (± SD) and range) for claws of female bearded and ringed seals by period. Note, these concentrations are not directly comparable between species because claw homogenizing methods differed. The method used on ringed seal claws increased progesterone extraction efficiency by 1.5 times over the method used on bearded seal claws (Karpovich *et al.* 2020)

Species	Period	Cortisol mean ± SD (pg/mg claw)	Cortisol range (pg/mg claw)	Progesterone mean ± SD (pg/mg claw)	Progesterone range (pg/mg claw)
Bearded seal	Pre-1968	13.1 ± 8.3	2.6–49.1	52.4 ± 20.5	9.2–123.9
Bearded seal	Post-1998	20.8 ± 18.2	2.0–154.8	53.3 ± 24.8	4.4–320.4
Ringed seal	Pre-1968	6.1 ± 2.1	2.8–17.0	133.3 ± 37.1	39.1–232.9
Ringed seal	Post-1998	8.0 ± 3.3	3.5–28.6	130.2 ± 35.1	44.4–256.4

**Table 3:** δ^13^C (Suess corrected) and δ^15^N (mean (± SD) and range) for claws of female bearded and ringed seals by period

Species	Period	δ^13^C mean (± SD)	δ^13^C range	δ^15^N mean (± SD)	δ^15^N range
Bearded seal	Pre-1968	-14.7 ± 0.5	-16.8 – -13.9	15.4 ± 0.6	13.6–16.9
Bearded seal	Post-1998	-15.5 ± 0.7	-17.6 – -14.5	15.9 ± 0.7	13.9–17.9
Ringed seal	Pre-1968	-15.6 ± 0.5	-16.8 – -14.7	17.5 ± 0.6	14.5–19.1
Ringed seal	Post-1998	-17.2 ± 1.2	-20.3– -15.6	17.1 ± 0.8	13.9–19.4

### Species comparison

Due to the different homogenization methods used on the claws, bearded seal hormone concentrations were expected to be lower than ringed seal concentrations and female bearded seals had significantly lower progesterone concentrations than female ringed seals (Table 2, F = 34.3, *P*  < 0.05, F = 118.2, *P* < 0.05, respectively). However, female bearded seals had significantly higher cortisol concentrations, suggesting that this difference would be even higher if homogenization methods were the same. There was a significant difference in δ^13^C and δ^15^N between species (F = 24.4, *P*  < 0.001, F = 57.1, *P*  < 0.001, respectively), where female ringed seals were more negative in δ^13^C and higher in δ^15^N (Table 3).

### Pregnancy determination by progesterone

**Figure 4:**
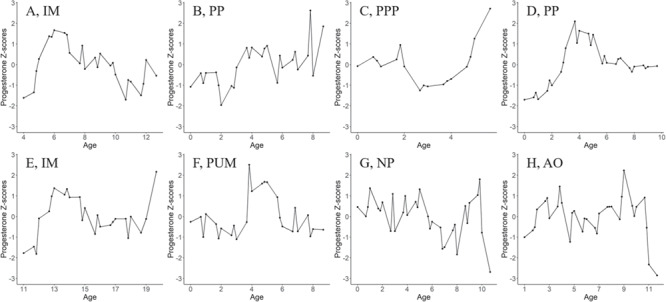
Progesterone Z-score by age of selected individual bearded (top) and ringed (bottom) seals. The oldest age of each animal corresponds to P, or the proximal claw band (closest to the body of the seal) and is the last point in each graph. The second to last point in these graphs is D, the band laid down in the season before the seal was harvested. **A.** An implanted (IM) bearded seal, **B.** a post-partum (PP) bearded seal, **C.** a primiparous, post-partum (PPP) bearded seal, **D.** a post-partum (PP) bearded seal, **E.** an implanted (IM) ringed seal, **F.** a primiparous, unimplanted (PUM; i.e. embryonic diapause) ringed seal, **G.** a non-pregnant (NP) ringed seal and **H.** an anovulatory (AO) ringed seal.

**Figure 5:**
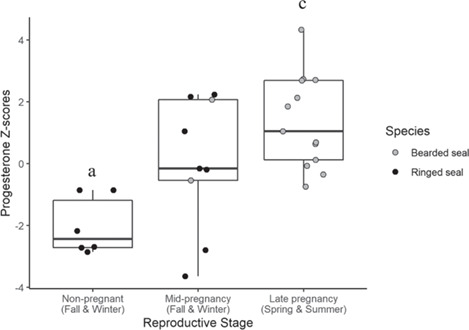
Progesterone Z-scores by pregnancy stage (which considers the reproductive status and season of harvest) for female bearded and ringed seal claws. Because it cannot be determined whether unimplanted seals have a blastocyst, are pseudopregnant, ovulating, or non-pregnant they were excluded from this analysis (*n* = 2 which were noted as unimplanted in the summer). The horizontal line within each box indicates the median, the box encompasses 25–75% of the data and the vertical lines (whiskers) include 5–95% of the data. Progesterone Z-scores were significantly different by reproductive stage (ANOVA, F = 6.5, *P* = 0.002). Non-pregnant seals (*n* = 6, a) have significantly lower progesterone Z-scores than seals in late pregnancy (*n* = 13, c; Tukey multiple comparison of means, *P* = 0.002). Seals in mid-pregnancy (*n* = 9), implanted seals harvested in fall or winter, were not significantly different from either non-pregnant or late-pregnancy seals (Tukey multiple comparison of means, *P* = 0.11, *P* = 0.26, respectively).

**Figure 6:**
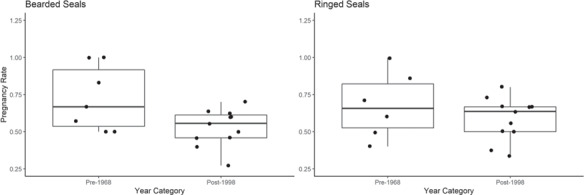
Pregnancy rate of individual seals based on progesterone Z-scores of claw annuli for bearded and ringed seals by pre-1968 and post-1998 periods. Pregnancy rate was calculated by summing a maximum of one light band per year with a progesterone Z-score above 0, divided by the number of years after which the seal was first pregnant in the claw to the year of harvest. Due to the method of calculating pregnancy rate, primiparous individuals and individuals harvested at age four or younger were not included in this analysis. Bearded seals show significantly higher pregnancy rates pre-1968 (*n* = 7 seals, *n* = 30 light bands) than post-1998 (*n* = 11 seals, *n* = 52 light bands, ANOVA, F = 5.9, *P* = 0.03), and ringed seals show no difference between pre-1968 (*n* = 6 seals, *n* = 24 light bands) and post-1998 (*n* = 11 seals, *n* = 53 light bands ANOVA, F = 1.1, *P* = 0.31).

We expected pregnant (i.e. implanted) and newly post-partum seals would show an increase in progesterone from D to P (from second most to most recently deposited) bands, while anovulatory and non-pregnant seals would decrease or be the same (Table 4, Fig. 4). Progesterone Z-scores decreased slightly for all implanted bearded seals from D to P (100%, or 3 of 3), but D progesterone Z-scores were much higher than the previous band (Fig. 4A). Progesterone Z-scores increased for a majority (75%, or 9 of 12) of post-partum bearded seals when comparing D to P (e.g. Fig. 4B-4C, example of an exception Fig. 4D). For ringed seals, a majority of implanted females exhibited increased progesterone Z-scores from D to P (66%, or 4 of 6; e.g. Fig. 4E), but unimplanted females decreased or stayed the same from D to P (e.g. Fig. 4F). Non-pregnant and anovulatory females decreased in progesterone Z-score from D to P (Fig. 4G-4H). Comparing progesterone Z-scores of the most proximal bands (P) by reproductive category revealed mean progesterone Z-scores ± SD for non-pregnant seals as −2.0 ± 0.9, unimplanted as 0.6 ± 1.7, implanted as 0.0 ± 2.0 and post-partum as 1.5 ± 1.4. We found that non-pregnant seals (*n* = 5) harvested in fall and winter had significantly lower progesterone Z-scores compared to late pregnancy seals (pregnant and post-partum seals harvested in spring and summer; *n* = 13, ANOVA, F = 6.5, *P* = 0.002, Tukey multiple comparisons of means, *P* = 0.002; Fig. 5), but mid-pregnancy individuals (pregnant individuals harvested in the fall and winter) were not significantly different than non-pregnant or late pregnancy seals (*n* = 9, ANOVA, F = 6.5, *P* = 0.002, Tukey multiple comparisons of means, *P* = 0.11; Fig. 5). Bearded seal pregnancy rates of sampled seals in this study were significantly higher pre-1968 (*n* = 7 seals, *n* = 30 light bands) compared to post-1998 (*n* = 11 seals, *n* = 52 light bands, ANOVA, F = 5.9, *P* = 0.03). Ringed seals show no difference in pregnancy rate by period (pre-1968: *n* = 6 seals, *n* = 24 light bands, post-1998: *n* = 11 seals, *n* = 53 light bands, ANOVA, F = 1.1, *P* = 0.31, Fig. 6).

**Table 4:** Progesterone concentration mean (± SD) (pg/mg claw), range, progesterone Z-score mean (± SD), range and a description of the difference from the second most (D) to most recently deposited (P) bands. The expectation is that seals which are unimplanted, implanted and post-partum would show an increase in concentration from D to P, while non-pregnant and anovulatory seals would decrease or be the same. NA indicates there was no, or too few, data to calculate the mean (e.g. for non-pregnant and unimplanted bearded seals)

Species	Pregnancy status	Sample size	[Progesterone] mean (± SD)	[Progesterone] range	Progesterone Z-score mean (± SD)	Progesterone Z-score range	Difference from D to P
Bearded seal	Non-pregnant	0	NA	NA	NA	NA	NA
	Unimplanted (early pregnancy)	1	153.4	NA	3.3	NA	Increase
	Implanted (mid-pregnancy)	3	66.7 ± 48.7	93.5–217.1	0.26 ± 1.6	-0.7–2.1	Decrease from high point
	Postpartum (late-pregnancy)	12	76.1 ± 25.4	42.9–123.9	1.5 ± 1.42	-0.3–4.3	9 of 12: Increase
	Anovulatory	0	NA	NA	NA	NA	NA
Ringed seal	Non-pregnant	3	84.9 ± 41.9	111.7–147.6	-1.9 ± 0.95	-2.7 – -0.9	Decrease
	Unimplanted (early pregnancy)	4	126.5 ± 15.6	93.5–217.1	-0.07 ± 0.98	-11–0.8	3: same, 1: decrease
	Implanted (mid-pregnancy)	6	159.6 ± 48.7	93.5–217. 1	0.39 ± 1.9	-2.8–2.2	4 of 6: Increase
	Postpartum (late-pregnancy)	0	NA	NA	NA	NA	NA
	Anovulatory	4	82.0 ± 15.8	65.7–103.7	-2.5 ± 1.2	-3.6 – -0.9	Decrease or same

### Relationships among progesterone, cortisol and stable isotopes

Post-1998, progesterone Z-scores and cortisol Z-scores did not differ by region or species. Therefore, we combined regions when analyzing these data for both pre-1968 and post-1998. For bearded seals pre-1968, progesterone Z-scores exhibited a positive relationship with both δ^15^N and age (t = 3.4, *P* = 0.001 and t = 2.4, *P* = 0.016, respectively). Post-1998 progesterone Z-scores exhibited a negative relationship with δ^13^C (t = −4.0, *P* < 0.001). For ringed seals pre-1968, progesterone Z-scores had a negative relationship with δ^13^C (t = −2.3, *P* = 0.022). However, post-1998 there was no relationship between progesterone Z-scores and stable isotopes or age (see Fig. S4 for summary of relationships for parameters which influence progesterone).

**Figure 7:**
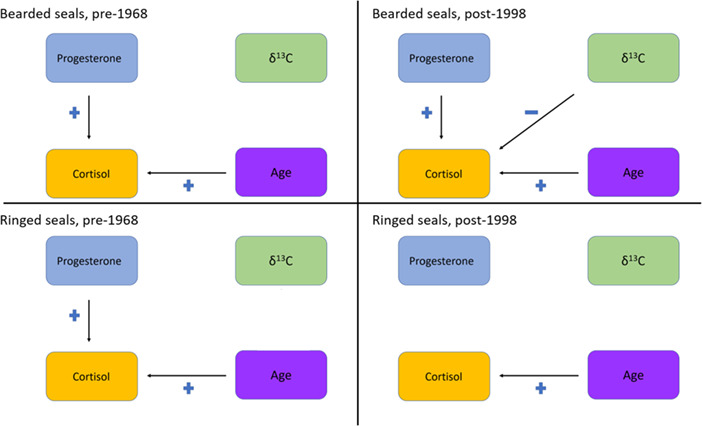
Results of relationships between cortisol Z-scores, average progesterone Z-scores, δ^13^C, by species period (1953–1968 and 1998–2014)

Female bearded seals pre-1968 cortisol Z-scores had a positive relationship with age and average progesterone Z-score (t = 4.6, *P* < 0.001 and t = 3.1, *P* = 0.003, respectively). Bearded seal cortisol Z-scores during post-1998 had a positive relationship with age and average progesterone Z-score; but a negative relationship with δ^13^C (t = 6.8, *P* < 0.001, t = 3.4, *P* = 0.001, t = −3.4, *P* = 0.001, respectively). For ringed seals pre-1968, cortisol Z-scores had a positive relationship with age and average progesterone Z-score (t = 3.6, *P* = 0.001 and t = 3.2, *P* = 0.002, respectively), but post-1998 cortisol Z-scores only had a positive relationship with age (t = 7.4, *P* < 0.001; relationships for parameters which influence cortisol are summarized in Fig. 7).

A preliminary analysis was carried out on annual claw growth (see Supplementary Information) to explore how hormones and stable isotopes vary with annual claw growth as annual claw growth may associate with body condition (Wittmann *et al.* 2016; Nguyen *et al.* 2017; Boucher *et al.* 2020). Annual claw growth in pre-1968 bearded seals exhibited a significant positive association with δ^13^C (t_1,71_ = 2.3, *P* = 0.03) and progesterone Z-scores (t_1,71_ = 2.4, *P* = 0.02) and a negative association with age (t_1,71_ = −4.7, *P*  < 0.001). No relationships in annual claw growth were found for bearded seals post-1998 or for ringed seals during either period.

## Discussion

### Progesterone as an indicator of pregnancy

Progesterone concentrations in claw annuli from female bearded and ringed seals were used to assess pregnancy rates up to 14 and 12 years prior to death in this study, respectively. Progesterone concentration was significantly higher in ringed seals, though this was an artifact of differences in claw homogenization methods (Table 2). Progesterone Z-scores matched the physiological expectation that non-pregnant females would have the lowest progesterone Z-scores, followed by unimplanted females, then implanted females, with post-partum females (representative of late pregnancy) having the highest progesterone Z-scores (Boyd 1991). The difference between non-pregnant and late pregnancy females was significant (Fig. 5). Once mature, most bearded and ringed seals ovulate and are capable of pupping annually (Frost and Lowry 1981; Crawford *et al.* 2015); however, some early pregnancies will terminate before parturition. For example, up to 40% of early pregnancies are known to terminate before implantation in humans (Jauniaux and Burton 2005). The mean progesterone Z-score of zero for unimplanted females supports that, on average, ringed and bearded seals are ovulating, and could be pregnant, every year. There are exceptions as was seen with the two anovulatory ringed seals, but this is uncommon (ADF&G unpublished data). Furthermore, although otariids exhibit an increase in progesterone during diapause, it is not known whether phocids do (Atkinson 1997; Renfree and Fenelon 2017). Due to these unknowns, five unimplanted ringed seals were not included in the analyses.

In this study, bearded seals exhibited a significantly higher pregnancy rate pre-1968 (0.72 ± 0.22, *n* = 7 seals) than post-1998 (0.53 ± 0.12, *n* = 11 seals, Fig. 6), which is in contrast to data from Quakenbush *et al.* (2011b) where pregnancy rate was lowest in the 1960s (88.3%, *n* = 163 seals) and higher in the 2000s (93.9%, *n* = 66 seals). In this study, ringed seal pregnancy rate did not significantly differ pre-1968 (0.68 ± 0.22, *n* = 6 seals) as compared to post-1998 (0.58 ± 0.15, *n* = 11 seals, Fig. 6), which is similar to data from Quakenbush *et al.* (2011a) where pregnancy rate was lowest in the 1960s (76.8%, *n* = 354 seals) and slightly higher in the 2000s (79.5%, *n* = 44 seals). Pregnancy rates from Quakenbush *et al.* (2011a, 2011b) were inclusive of unimplanted seals, which do not always result in pregnancy. This calculation, then, provides the percentage of seals that had ovulated out of all sexually mature females, or, that is, ovulation rate rather than pregnancy rate. Crawford *et al.* (2015) showed no statistical difference in bearded and ringed seal ovulation rates when comparing 1975–1984 and 2003–2012 using the same methods. It is possible that fewer bearded seals carried pups to term after ovulating in the post-1998 dataset. The data available later in gestation from the claw could more closely determine pregnancy rate. Although this study shows progesterone can be used in claws to identify pregnancies during an individual’s lifetime, the sample size of individual seals in this current study is too small, or the females sampled were not representative, to determine annual pregnancy rates at a population level relative to a large harvest sampling program (Quakenbush *et al.* 2011a, 2011b; Crawford *et al.* 2015)*.*

### Cortisol as an indicator of stress

The bearded seals exhibited higher cortisol concentrations than ringed seals despite different methods for claw powder homogenization was unexpected (Table 2). Bearded seals also had higher whole whisker cortisol concentrations compared to ringed seals (Fig. S5). Bearded seals may have naturally higher cortisol concentrations than ringed seals, they could have a more active HPA axis in response to physical or psychological stress, or other unknown factors. Because cortisol is associated with many physiological processes beyond the stress response (MacDougall-Shackleton *et al.* 2019) we are cautious to interpret this difference between these species as stress related (i.e. that bearded seals experience ‘more’ stress than ringed seals).

Bearded seals showed an increase in cortisol concentration from pre-1968 to post-1998, but ringed seals did not (Table 2). To understand what environmental factors may influence cortisol during different periods, we explored how sea ice coverage, which declined 33–38% during 1953–2006 increasing to 47–57% during 1979–2006 (Stroeve *et al.* 2007), and diet, shown to shift to more fish (benthic and pelagic) in 1975–1984 as compared to 2003–2012 (Crawford *et al.* 2015), may affect cortisol in bearded but not ringed seals.

The reduction in sea ice is predicted to promote pelagic over benthic production in the southeastern Bering and Chukchi seas (Hunt Jr *et al.* 2002; Grebmeier *et al.* 2006). Although bearded seals feed on more benthic prey than ringed seals (Burns 1981), and thus would potentially be affected more by the predicted change, there is no evidence that a change in diet has negatively affected bearded seals. The diet of both seal species has been dominated by more fish (benthic and pelagic) and less invertebrate prey (benthic and pelagic) in recent years (Crawford *et al.* 2015). Ringed seals seem to have responded well to this recent change in diet indicated by faster growth, thicker blubber and decreased age of sexual maturity (Crawford *et al.* 2015), which is consistent with our finding of no difference in ringed seal claw cortisol concentration between the two periods in our study. Although bearded seals consumed more fish (mostly benthic sculpin, cod and flatfish) in the 2000s as compared to the past (Crawford *et al.* 2015), no negative trends in growth rate, blubber thickness or age at sexual maturity have been detected, which may indicate the rise in cortisol for bearded seals from pre-1968 to post-1998 has little to do with a change in prey. However, a difference in diet due to environmental change in the Arctic, regardless of whether it is benthic or pelagic based, could contribute to an increase in cortisol concentration if the change in prey required some physical or physiological adjustments.

### Carbon and nitrogen stable isotopes as indicators of diet

Overall, carbon and nitrogen stable isotopes were consistent with what is known about diet of both species, and as expected bearded seals have less negative δ^13^C and lower δ^15^N than ringed seals because they are generalist benthic feeders at lower trophic levels, whereas ringed seals feed more pelagically (Cameron *et al.* 2010; Newsome *et al.* 2010; Crawford *et al.* 2015; Fleming *et al.* 2018).

For both bearded and ringed seals δ^13^C became significantly more negative from pre-1968 to post-1998, whereas there was no difference in δ^15^N for either seal species when comparing these two periods. A more negative δ^13^C could indicate that both species are eating more pelagic prey and/or feeding farther offshore during the more recent period, are consuming a more diverse diet overall and/or are relying more on their blubber stores (Newsome *et al.* 2010; Fleming *et al.* 2018). While there is no evidence that blubber thickness declined for either species, both species are consuming more fish and the same, or fewer, invertebrates than in the past (Crawford *et al.* 2015). The shift to more and a greater diversity of fish in the diet could support our results for more negative δ^13^C in bearded and ringed seal claws from pre-1968 to post-1998. Specifically, ringed seals consumed a more diverse diet of fish: five dominant fish species in the 2000s as compared to three in the 1960s and 1970s (Quakenbush *et al.* 2011a), which could explain how for ringed seals the range of δ^13^C more than doubled from pre-1968 (−20.3 to −15.6‰, 4.7‰) compared to post-1998 (−16.8 to −14.7‰, 2.1‰). This is consistent with a wider niche as Boucher *et al.* (2020) also showed for ringed seals in the Beaufort Sea.

However, Carroll *et al.* (2013) found that δ^15^N differed between bearded and ringed seal claws during 1998–2010, whereas δ^13^C did not. This could be due to a few outliers in each dataset. In this study, the ice seals with more negative mean δ^13^C included the only bearded seal from Shishmaref (Chukchi Sea), the only ringed seal from Utqiaġvik (formerly Barrow, near the border of the Chukchi and Beaufort seas) and one of two ringed seals from Gambell (Bering Sea). The ringed seal with significantly higher mean δ^15^N was from Shishmaref. Many phocid species have individuals which are feeding specialists, even if the species is characterized as generalists (Hindell *et al.* 2012). Furthermore, the seals in Carroll *et al.* (2013) were primarily from the Chukchi Sea, whereas seals sampled in this study represented the Bering and Chukchi seas. Thus, specialist foragers and different foraging regions could explain the difference in δ^13^C and δ^15^N in bearded and ringed seals between our study and Carroll *et al.* (2013).

### Relationships among progesterone, cortisol and stable isotopes

Overall, for both bearded and ringed seals, there are positive associations with averaged progesterone Z-scores and cortisol Z-scores and with cortisol Z-scores and age. The positive association of progesterone Z-scores and cortisol Z-scores is likely due to the increase of cortisol during pregnancy to support fetal development, exhibited in many mammalian species (Foley *et al.* 2001; Dloniak *et al.* 2006; Hunt *et al.* 2006; Edwards and Boonstra 2018; Heimbürge *et al.* 2019). Surprisingly, ringed seals post-1998 did not show the same associations between cortisol Z-score and progesterone Z-score. This may be due to the timing of harvest relative to when the hormones are deposited in the claw tissue. Additionally, of the 11 ringed seals harvested post-1998, two were anovulatory without any visible CL or CA, an extremely rare occurrence only seen in 2011 (ADF&G unpublished data). The occurrence of anovulatory seals could be linked to the increase in sick and moribund seals stranded on the northwest coast of Alaska in 2011 and documented as an Unusual Mortality Event (UME; Burek-Huntington *et al.* 2012). With an increase in stranded animals again in 2019, there is potential for further investigation into this phenomenon.

An increase in cortisol as mammals age is common and related to older animals taking a longer time to return to their baseline after activation of the HPA axis, which may explain the association among cortisol Z-scores and age in bearded and ringed seals (Reeder and Kramer 2005; Hunt *et al.* 2006). The distal, more worn, end of the claws could also be reduced in cortisol due to the ‘washing out’ of cortisol from portions of the claw exposed to the environment for longer periods of time (Kirschbaum *et al.* 2009), also noted as a possibility by Karpovich *et al*. (2018) in phocid whiskers and possible for hair (Kirschbaum *et al.* 2009; Hamel *et al.* 2011; Li *et al.* 2012). However, there has been conflicting information about washout of cortisol (Sharpley *et al.* 2012). For instance, hair follicles can independently synthesize and secrete cortisol (Ito *et al.* 2005), therefore, cortisol could be included at the root of claws from sources other than direct deposition from circulation during claw growth. In this study, neither bearded or ringed seal claws exhibited a change in mean progesterone concentration from pre-1968 to post-1998 and ringed seal claws showed no difference in mean cortisol concentration from pre-1968 to post-1998. Bearded seal claws did show a difference in mean cortisol concentrations, where cortisol concentrations were lower pre-1968 (older museum stored claws) as compared to post-1998 (short-term storage; Table 2). While this could be a biological difference, it is possible that long term storage of bearded seal claws may impact the samples. However, Bechshøft *et al.* (2012) showed mean cortisol concentrations in museum hides had higher mean cortisol concentrations than hides from recently deceased animals. Therefore, this suggests that cortisol and progesterone are stable in historic museum samples. We assumed that washout did not affect hormone concentrations along the length of the claws; however, we acknowledge that hormones may have been lost from the claw over time.

Pre-1968 bearded seal progesterone Z-scores exhibited a negative relationship with δ^15^N and age. Increasing progesterone with decreasing δ^15^N, indicates pregnant females are feeding at a lower trophic level, or possibly exploiting more resources while pregnant (Newsome *et al.* 2010; Fleming *et al.* 2018). Furthermore, during this time, younger adults were associated with higher progesterone, which could indicate that younger bearded seal females were more fecund, although decreases in reproductive success in older females, associated with reproductive senescence, have not been documented in bearded seals (Crawford *et al.* 2015). Therefore, a larger number of young females during the pre-1968 period could explain the increased pregnancy rate during this time, but pre-1968 seals were only assigned a minimum age based on claw banding, which makes this difficult to assess. Bearded seals post-1998 exhibited a negative relationship between δ^13^C and progesterone: as progesterone Z-scores increased, δ^13^C became more negative. This result suggests that pregnant bearded seals (those with the highest progesterone Z-scores) may change their diet or foraging region to consume more pelagic or off-shore prey compared to non-pregnant seals (Newsome *et al.* 2010; Fleming *et al.* 2018). Pre-1968 ringed seals similarly exhibited a negative relationship between progesterone Z-scores and δ^13^C, however post-1998 ringed seals showed no relationships between progesterone Z-scores and δ^13^C, δ^15^N or age. This may be further support for the plasticity of ringed seals and their ability to successfully exploit available resources attributing to their successful persistence in the Arctic. Overall, changes in carbon stable isotopes from pre-1968 to post-1998, without changes in progesterone concentrations between these periods, suggests the ringed seal’s ability to maintain reproductive capacity while adjusting to a changing prey structure, which is likely driven by environmental change (Quakenbush *et al.* 2011a; Crawford *et al.* 2015). Bearded seals in the present study, on the other hand, exhibited a significant decrease in mean pregnancy rate of all sampled females from pre-1968 to post-1998 without accompanying changes in progesterone concentration (likely due to how progesterone Z-scores account for difference in mean progesterone concentration baselines of individuals); this decrease in pregnancy rate, however, could be due to low sample size. There was a positive association between δ^13^C and cortisol, which suggests the changes in carbon stable isotopes (i.e. diet) and cortisol may affect bearded seal reproductive capacity, however other factors not studied may be involved (e.g. density dependence).

## Conclusion

Bearded and ringed seal claws store progesterone, cortisol and carbon and nitrogen stable isotopes over the span of claw growth, an average of *ca.* 9 years. Progesterone in claws can be used to identify the number of pregnancies and pregnancy rate in individuals over the span of claw growth in both species, however calculating pregnancy rates per year or comparative period will require larger sample sizes of a representative sample of the population. Bearded seal claws increased in cortisol concentration from pre-1968 to post-1998, which is not paralleled in ringed seals, suggesting a different response to environmental change. Carbon and nitrogen stable isotopes in claws mirror stomach content observations and known diet and became more negative over time in both species. Finally, cortisol is positively associated with progesterone and age for both species no matter the time span, except for ringed seals post-1998 which was the only category for which anovulatory ringed seals were harvested. Combining hormone and stable isotope values has added to our understanding of the biology of bearded and ringed seals. These values can be used in conjunction with other studies to track trends in progesterone, cortisol and stable isotopes to evaluate reproduction, stress and diet changes as well as the connection among these life history parameters through time.

## Data availability

Data and R code used for this manuscript has been made available with Sea Open Scientific Data Publication (SEANOE): https://doi.org/10.17882/77352.
